# Clinical features and histopathological analysis of corneal myxoma

**DOI:** 10.1038/s41598-022-15475-1

**Published:** 2022-07-01

**Authors:** Bader S. Al-Qahtani, Hind M. Alkatan, Rajiv Khandekar, Ahmed Al-Salem, Samar A. Al-Swailem

**Affiliations:** 1 Research Department, King Khaled Eye Specialist Hospital, PO Box 7191, Riyadh, 11462 Saudi Arabia; 2 Ophthalmolmology Department, King Faisal Specialist Hospital, Riyadh, Saudi Arabia; 3 Ophthalmology Department, College of Medicine, King Saud University, Riyadh, Saudi Arabia; 4 King Saud University Medical City, College of Medicine, King Saud University, Riyadh, Saudi Arabia

**Keywords:** Diseases, Medical research, Pathogenesis

## Abstract

To describe the presentation, histopathological characteristics, and management outcomes for corneal myxoma. This one-armed cohort study evaluated histologically confirmed consecutive cases of corneal myxoma. Data were evaluated on demographics, clinical presentation, management, histopathological and immunohistochemical features, and outcomes; visual acuity and corneal clarity. The study sample was comprised of 10 eyes (10 patients). The median age at presentation was 10.5 years. Five eyes had high intraocular pressure, four eyes had decreased distance visual acuity and one eye became discolored. Surgical management included penetrating keratoplasty (8 eyes), phototherapeutic keratectomy (1 eye), and evisceration because of a blind painful eye (1 eye). Postoperative best-corrected distance vision ranged from 20/20 to 20/60 (1 eye), < 20/60 to 20/200 (2 eyes), < 20/200 to 20/400 (1 eye), < 20/200 to light perception (4 eyes) and no light perception (1 eye). The histopathology of these lesions showed typical subepithelial proliferating spindle-shaped cells of mesenchymal origin within a myxoid stroma rich in glycosaminoglycan. The median duration of follow-up was 5 years. Recurrence was observed in an eye that underwent local excision. Corneal myxoma is a rare lesion that is presumably isolated, secondary, and reactive in nature. Surgically management yields reasonably favorable outcomes.

## Introduction

Myxomas are benign tumors that are irregularly shaped with a jelly-like consistency. They originate from modified fibroblasts and myofibroblasts that produce unusually small quantities of collagen and copious amounts of glycosaminoglycans (rich in hyaluronic acid). These lesions are usually not capsulated^1^.

Ocular myxomas have been identified in the eyelids, conjunctiva, cornea, and more recently, in the orbit^2^. Individual cases have been reported in the literature^3^. Primary corneal myxoma is extremely rare. Secondary corneal myxomas develop as a reactive process, arising from corneal diseases (e.g., infective keratitis, keratoconus, and bullous keratopathy) or after trauma or surgery (e.g., repeated excimer laser in an edematous cornea) or injury-inducing chronic corneal edema^3–6^.

To the best of our knowledge, 21 cases have been published so far ^3–19^. The largest published series documents 6 cases^4^. All the previous reports in the literature indicate the benign nature of this lesion.

In this study, we present the clinical profile, tissue findings and the surgical management of 10 patients with corneal myxoma diagnosed and managed for 15 years at a tertiary eye hospital in central Saudi Arabia.

## Methods and patients

The study was performed in accordance with the Tenets of the Declaration of Helsinki. The Institutional Review Board committee of the King Khaled Eye Specialist Hospital (KKESH) approved this retrospective research (1461-CR) study. This committee is related to the hospital in which the study was performed & the committee supervises all research activities in the hospital. Informed written consent was obtained from all 10 patients (or parents/guardian) for the purpose of using photos in this scientific publication & participating in the study. No monetary incentivization of patients for recruitment and retention was given.

This one-armed retrospective cohort study included all histologically confirmed cases of primary and secondary corneal myxoma at a tertiary eye hospital (KKESH), Riyadh, Saudi Arabia between January 2000 and December 2015. All cornea cases at KKESH are usually referred by physicians from all cities in Saudi Arabia. We defined primary corneal myxoma as a histologically confirmed lesion with no prior history of ocular surgery or trauma. Secondary corneal myxomas were defined as those with a prior history of ocular trauma, surgery or corneal pathology.

Data were collected on patient demographics such as age, gender and the affected eye. The best-corrected distance visual acuity (BCVA) was measured at a 6-m distance using a projector. If vision testing was not possible at 6-m, it was tested as counting fingers at 3 m with the ability to detect the direction of light. Preoperative and postoperative examinations and photographs were taken with a slit lamp biomicroscopy (Haag-Streit Group, Bern, Switzerland). Intraocular pressure (IOP) was measured with a Tonopen (Medtronic Inc., Dublin, Ireland).

All cases were treated by either penetrating keratoplasty (PKP) or phototherapeutic keratectomy (PTK) for deep and superficial lesions respectively. Senior members of the Cornea unit performed these surgeries in the major operating theater under strict aseptic technique and following standard techniques to reduce inter-operator variability. Imported corneal donor tissue was used for these procedures that met the Eye Bank Association of America (EBAA) requirements. Consent for surgeries under general anesthesia was taken from the guardian for patients below the age of 18 years, and consent under local anesthesia was taken directly from elder patients. After PKP surgery, all patients were treated with topical prednisolone acetate 1% (Pred Forte; Allergan Inc., Dublin, Ireland) for a minimum of 6 months. Topical ofloxacin 0.3% (Optiflox; Jamjoom Pharma, Jeddah, Saudi Arabia) or moxifloxacin 0.5% QID (Vigamox; Alcon Inc., Fort Worth, TX, USA) were prescribed for 2–3 weeks. The postoperative examination was scheduled at 1 day, 1 week, 1 month, and every 3 months for 2 years and yearly thereafter. Patients with complications were assessed more frequently for appropriate management.

BCVA preoperatively and at the last postoperative visit was graded as excellent (20/20 to 20/60), moderate visual impairment (< 20/60 to 20/200), severe visual impairment (< 20/200 to 20/400), blindness (as per World Health Organization criteria; < 20/400), and no light perception (NLP).

The corneal buttons removed during surgery were preserved in formalin and then embedded in paraffin for routine processing and preparation of hematoxylin and eosin (H&E) staining as well as periodic acid-Schiff staining for histologic sectioning. Special stains performed included, alcian blue to highlight the myxomatous background of the lesions, and immunohistochemical stains such as vimentin, to confirm that the proliferating cells in the lesion were of mesenchymal origin, and smooth muscle actin (SMA). The same pathologist graded the intensity of staining as follows: 1 (mild staining) to 3 (marked staining). The cellularity of the lesions was also graded from 1 (hypocellular) to 3 (hypercellular). The slides were examined at high resolution with a binocular microscope (Optika Srl, Lombardy, Italy).

Data were collected on an Excel spreadsheet (Microsoft Corp., Redmond, WA, USA). Statistical analysis was performed using a statistical package for social sciences (SPSS 25; IBM Corp., Armonk, NY, USA). For qualitative variables, numbers and percentages were calculated. For quantitative variables, the median and interquartile range was calculated.

### Statement of ethics/studies involving human subjects

The study was approved by the Institutional Research Board (1461-CR) at King Khaled Eye Specialist Hospital. The tenets of the Declaration of Helsinki were followed at each step of the study.

### Informed consent

An informed written consent was obtained from all 10 patients (or parents/guardian) for the purpose of using photos in this scientific publication & participating in the study.

## Results

The study sample was comprised of 10 eyes of 10 patients (3 males, 7 females) with the corneal myxoma lesion involving seven right and three left eyes. The median age at presentation was 10.5 years [Interquartile Range (IQR) 1.8; 46.7] (range 1 year to 92 years). Four patients had an ocular history of surgery, trauma, and keratitis and were categorized as secondary-truly acquired-myxomas (Cases: 3, 4, 6, and 9). Another 4 had myxomas associated with developmental corneal disease including, Peter’s anomaly as a manifestation of anterior segment dysgenesis (ASD) and, these eyes were categorized as secondary-developmental-myxomas. Two patients had presumed primary myxomas with no diagnosis of ASD or previous corneal disease and/or intervention (Table 1). Five cases presented with high IOP, four of whom were < 15 years age at presentation. The median area of the lesion was 45.6 mm^2^ (IQR 30.0; 64.0) (range 8 mm^2^ to 70 mm^2^).

**Table 1:** Demographic and clinical presentation of patients with corneal myxoma.

Case no.	Age (years)	Gender	Primary vs secondary	Clinical presentation	Clinical appearance
1	2	Female	Secondary (developmental)	ASD, High IOP	Whitish opacification
2	8	Female	Secondary (developmental)	ASD, High IOP	Whitish opacification
3	92	Female	Secondary (acquired)	High IOP	Whitish opacification
4	17	Male	Secondary (acquired)	Decreased VA, keratoconus	Whitish opacification
5	43	Female	Primary	Decreased VA	Whitish opacification
6	48	Male	Secondary (acquired)	Decreased VA	Whitish opacification
7	1	Female	Secondary (developmental)	ASD, High IOP	Whitish opacification
8	3	Female	Secondary (developmental)	ASD, High IOP	Hydrops
9	33	Male	Secondary (acquired)	Decreased VA, Post Fungal Keratitis	Dome-shaped lesion
10	4	Female	Primary	Discoloration of eye	Whitish opacification

*IOP* intraocular pressure, *VA* visual acuity, *ASD* Anterior segment dysgenesis.

**Figure 1:**
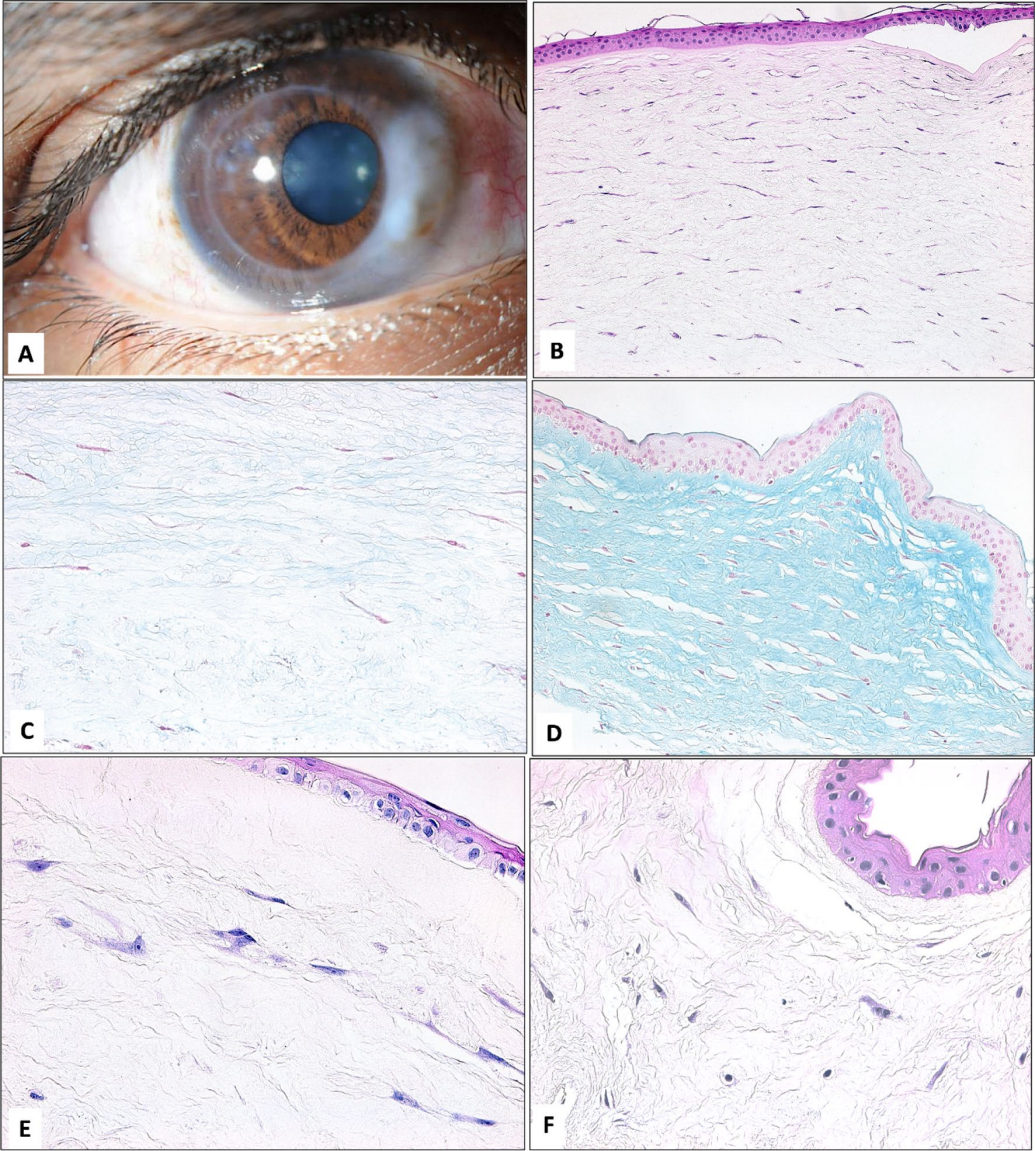
(**A**) The clinical appearance of the keratoconus in the right eye of a 17-years old male (Case 4) after PKP for corneal myxoma with an excellent outcome. There is a residual part of the myxomatous lesion at the recipient’s corneal rim adjacent to the corneal graft edge nasally. (**B**) The histopathological appearance of the corneal myxoma is composed of stellate and spindle-shaped cells anteriorly with thin corneal epithelium, bullae, and absent Bowman’s layer. Note the hypocellularity of the myxoma in this secondary type (× 200 magnification, Hematoxylin and Eosin). (**C**) Weak Alcian blue staining (< grade 1) in the same keratoconus case representing secondary-truly acquired-corneal myxoma (× 400 magnification). (**D**) Intense staining of Alcian blue (grade 3) of the primary myxoma in case 5 (× 200 magnification). (**E,F**) The histopathological appearance of primary myxoma in case 5 that was treated with LKP with intracellular epithelial edema, absent Bowmans’s layer and typical myxoma cells (× 400 magnification, Hematoxylin and Eosin).

Eight patients underwent PKP (Fig. 1a), one patient underwent local resection to debulk the mass and send a specimen to the histopathology lab followed by PTK, and one patient with a painful blind eye and high IOP underwent evisceration of the eye. One patient of those had PKP developed a graft failure. Another patient developed glaucoma that was managed surgically with a glaucoma drainage device. The patient who had local resection with PTK recurred within two months. Keratoplasty was offered to this patient, but he refused surgery. Fortunately, we could preserve most of the eyes. One child had a phthisical eye with controlled IOP using multiple anti-glaucoma medications. A 92-years old patient had an eye with uncontrolled IOP and pain and eventually underwent evisceration. The median duration of postoperative follow-up was 5 years (IQR 5; 6). The postoperative BCVA was 20/20 to 20/60 in one eye, < 20/60 to 20/200 in two eyes, < 20/200 to 20/400 in one eye, < 20/400 to light perception (LP) in four eyes and one eye had NLP. The eviscerated eye was not included in the grading of visual impairment.

**Figure 2:**
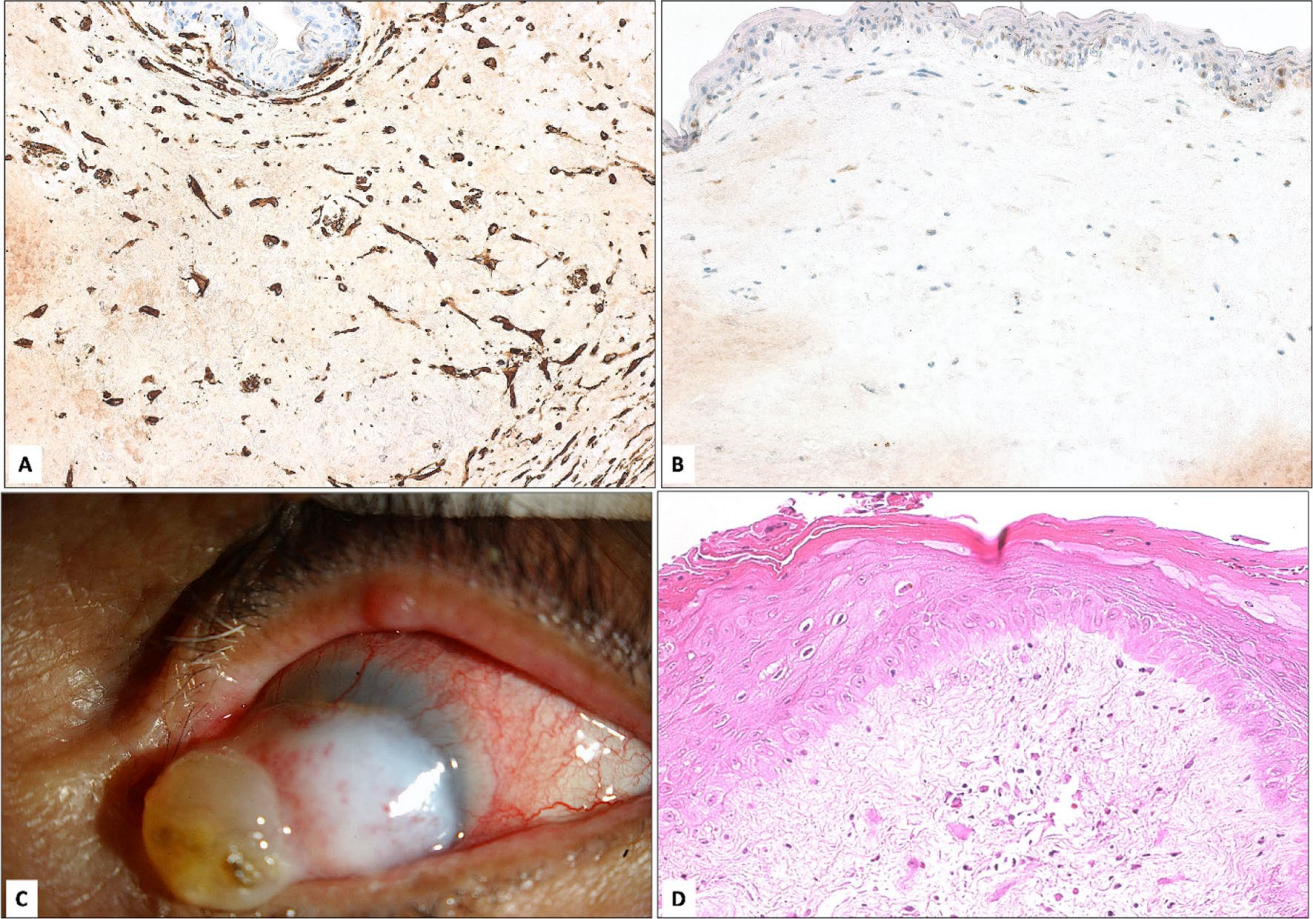
(**A**) The histopathological appearance of the positive reactivity of the spindle cells to the immunohistochemical staining for Vimentin in a typical secondary-truly acquired-corneal myxoma (Case 6) with an absent expression of Smooth muscle actin in (**B**) (× 400 magnification). (**C**) The clinical appearance of another secondary myxoma in an elderly female (Case 3) with epithelial hyperplasia and keratinization that was evident histopathologically in (**D**) the keratinized corneal epithelium above the myxoma (× 200 magnification, Hematoxylin and Eosin).

**Figure 3:**
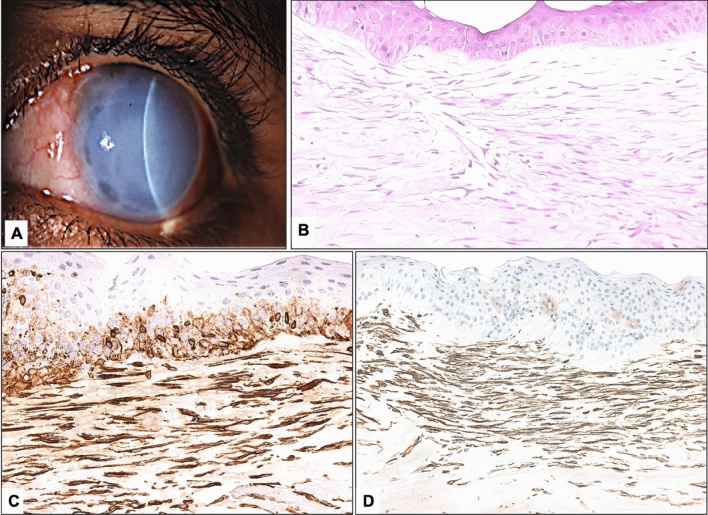
(**A**) The clinical appearance of case 7 with the diagnosis of Peter’s anomaly represents a case of secondary-developmental-myxoma (ASD-associated). (**B**) The corresponding histopathological appearance of the incidental associated corneal subepithelial myxoma shows absent Bowman’s layer and the typical spindle-shaped cells (× 400 magnification, Hematoxylin and Eosin). (**C**) The positive reactivity of the spindle cells of the same lesion to the immunohistochemical staining for Vimentin. (**D**) A similar reactivity to smooth muscle actin in contrast to the above secondary-truly acquired-myxoma (Case 6) with an absent expression of smooth muscle actin implicating different pathogenesis between the 2 groups (× 400 magnification).

**Figure 4:**
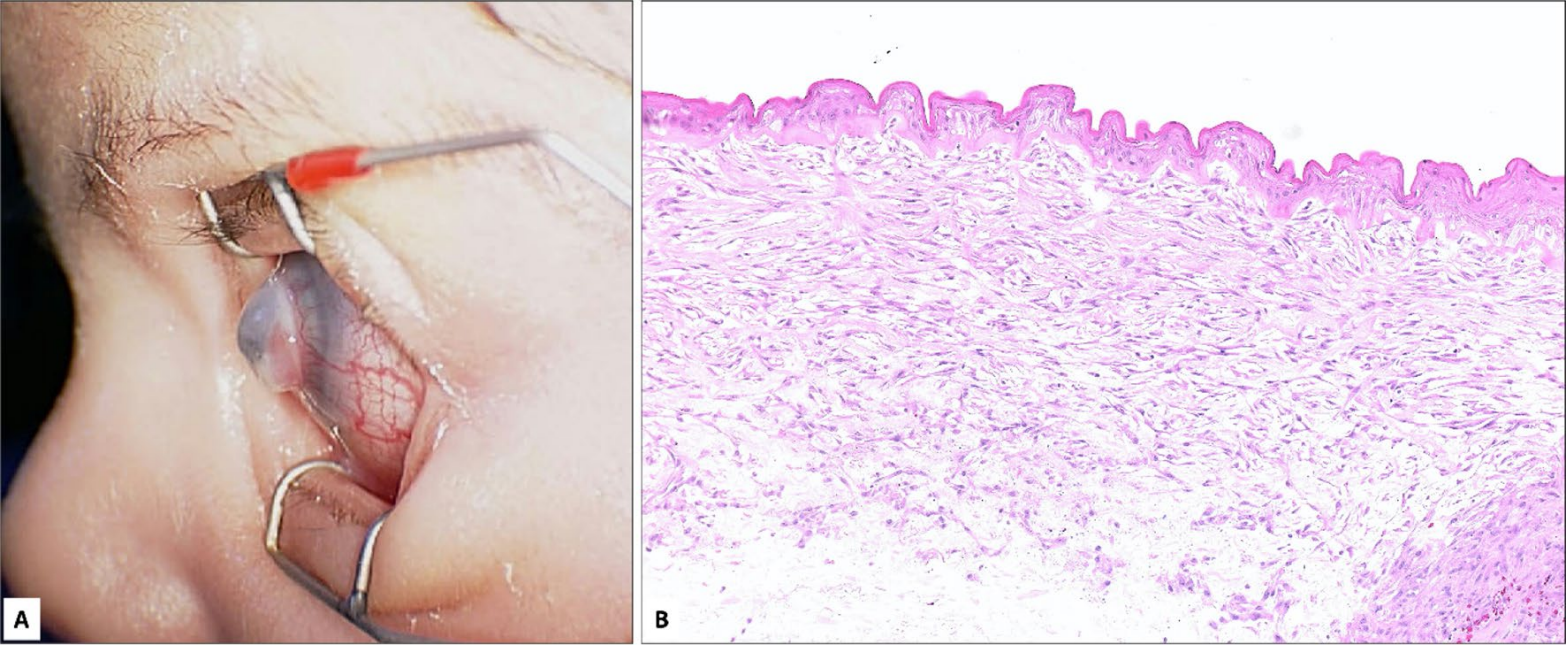
(**A**) Another case of Peter’s anomaly with an impressive looking myxoma clinically in case 8. (**B**) The histopathological appearance of the corneal subepithelial myxoma shows absent Bowman’s layer and the typical hypercellular spindle-shaped cells similar to the previous case in Fig. 3 (× 200 magnification, Hematoxylin and Eosin).

Table 2 presents the clinical features of all previously reported cases of corneal myxoma. Table 3 presents the histopathological, immunohistochemical, and clinical features of the study sample. The myxoma involved the corneal stroma with variable overlying epithelial changes mostly in the form of epithelial hyperplasia, irregularity with or without bullous changes, and intracellular edema. Nine out of 10 specimens shared the presence of epithelial edema either in the form of intracellular edema (5/10) or intraepithelial bullae (4/10). One case had epithelial thinning (in association with keratoconus) and another cornea showed evidence of epithelial metaplasia with keratinization. Bowman’s layer was absent in 9 (7 secondary and 2 primary myxomas) out of the 10 lesions and sub-totally absent above the myxoma in the remaining case. All lesions had spindle-shaped and stellate cells in a loose myxoid background rich in glycosaminoglycans (Fig. 1b) with variable Alcian blue staining between the secondary acquired myxoma in keratoconus and the primary myxoma case (Fig. 1c,d). In cases where a full-thickness corneal button was removed (8/10), Descemet’s membrane was centrally interrupted in all 4 cases with developmental abnormality in the cornea (ASD), while it was intact in 3/8 and showed artefactual interruption and detachment in one case of secondary myxoma that was associated with microbial keratitis (Case 9). The endothelium was attenuated in 7 out of 8 cases that underwent full-thickness corneal button removal. Almost all (9/10) cases showed strong reactivity of the tumor cells with vimentin stain (Figs. 2, 3) (one case had moderate staining). The 2 primary myxomas did not stain with SMA as expected. The secondary-developmental-myxomas exhibited this reactivity in 3 of 4 cases as demonstrated in Figs. 3 and 4 of Peter’s anomaly cases, while the secondary-truly acquired-myxomas showed reactivity with SMA antibodies in a single case of keratitis (1/4).

**Table 2:** Clinical features of all previous literature reports of corneal myxomas.

References	Sex	Age (year)	History	Appearance	Source of specimen
Mitvalksy ^15^	F	26	Keratitis, staphyloma resection	Soft, pedunculated, reddish mass	Enucleation
Bussy ^16^	M	66	Ulcer	Opaque and firm cornea, stromal vessels	Enucleation
Lo et al. ^12^	F	44	No ocular disease or trauma	Gelatinous, whitish mass	Superficial keratectomy
Pe ´rez-Grosmann et al. ^13^	M	57	Ulcer	White nodule	Evisceration
Le ´ger et al. ^19^	F	26	Keratoconus	Translucent, whitish mass	PKP
Wollensak et al. ^6^	M	48	Trauma, Scleral buckle, Lensectomy, Bullous Keratopathy	Whitish elevation	Superficial keratectomy
Hansen et al. ^8^	F	36	Strabismus surgery	Whitish, fleshy opacity	Superficial keratectomy
Robinson et al. ^3^	M	55	Trauma, multiple PTK	Gelatinous, whitish	PKP
Khan et al. ^7^	F	4 months	Peters’ anomaly	Hazy cornea, focal elevation	PKP
Alkatan et al. ^14^	M	58	Cataract surgery, pterygium removal	Whitish, elevated lesion	Lamellar Keratectomy
Soong et al. ^5^	M	65	No ocular disease or trauma	White gelatinous	Superficial keratectomy
Lang et al. ^17^	F	56	No ocular disease or trauma	Gelatinous, raised lesion	Superficial keratectomy
Peralta et al. ^18^	M	70	No ocular disease or trauma	Whitish nodule	Superficial keratectomy
Belliveau et al. ^4^ Case 1	M	45	Trauma, PKP	White, gelatinous elevation	Evisceration
Case 2	M	76	Trauma, cataract surgery	Glistening, whitish elevation	PKP
Case 3	M	69	Trauma, cataract surgery, scleral buckle	Opaque cornea, central thinning	Evisceration
Case 4	M	63	Trauma	White, gelatinous	Evisceration
Case 5	M	73	Trauma, PKP, cataract surgery	Whitish opacity	Lamellar biopsy
Case 6	M	80	PKP	White, gelatinous	Lamellar biopsy
Lim et al. ^10^	M	32	No ocular disease or trauma	Gelatinous mass and blisters	LKP
Chang et al. ^11^	M	70	No ocular disease of trauma	White, gelatinous elevation	Corneal biopsy

**Table 3:** The histopathological and immunohistochemical results of the corneas with clinical correlation.

Case No	Age (y)	Sex	Histopathology: Epithelium/Bowman’s layer/Histopathological Dx/DM: I = interrupted, N = Normal, I* artifacticious interruption			Cellularity (Hypo) equal to/less than 1–3 (Hyper cellular)	Stains grading 1–3			Clinical presentation
							Alcian B	Vimentin	SMA	
1	2	F	Hyperplasia Bullae/absent	ASD Peter’s	I	2	NA	3	1	High IOP
2	8	F	Hyperplasia Bullae/absent	ASD Peter’s	I	2	3	3	3	High IOP
3	92	F	Hyperplasia and keratinized /absent	Myxoma	N	1	0.5	3	0	High IOP
4	17	M	Thin, Bullae/interrupted	KC/hydrops	N	1	0.5	3	0	Decreased VA, keratoconus
5	43	F	Irregular/intra-cellular edema/absent	Myxoma (primary)	NA	2	3	3	0	Decreased VA
6	48	M	Intracellular edema/absent	Myxoma	NA	1	1	3	0	Decreased VA
7	1	F	Hyperplasia Large bullae /absent	ASD Peter’s	I	2	3	3	3	High IOP
8	3	F	Irregular/intra-cellular edema/absent	ASD Peter’s	I	3	3	2	0	High IOP
9	33	M	Irregular/intra-cellular edema/absent	Fungal K	I*	2	2	3	3	Decreased VA
10	4	F	Irregular/intracellular edema/absent	Sub-epithelial Myxoma (Primary)	N	1	3	3	0	Discoloration of eye

## Discussion

In our cohort, both primary and secondary corneal myxoma were histologically confirmed and had undergone surgical management. Although they were presented in the early stages and were managed by experienced surgeons, the visual outcomes were not very promising. One eye had to be removed, one case experienced recurrence, and one eye experienced graft failure. The histological findings of the current study on corneal myxoma concur with previous literature^5,7,10^.

Since our cohort included cases with histologically confirmed corneal myxoma, we could not report how many were clinically suspected, however, no preoperative diagnosis of myxoma was documented in the files of all cases until a histopathology report was presented. Clinically suspect corneal myxoma needs to be differentiated from nodular fasciitis, Salzmann’s nodular degeneration, foreign body reaction, pannus, corneal amyloid, contact lens-induced epithelial microcysts or corneal pseudocysts, corneal keloid, dermoid tumor, corneal squamous cell carcinoma and sarcoma^5,7,10^. In many cases, it is confirmed following excision and histological review as in the current study. Histological confirmation of the lesion (by incisional or excisional biopsy) is essential to distinguish myxoma from other lesions and affects further management of the myxoma as it determines the potential cellular origin, establishes the benign nature of the lesion, and helps with treatment and prognostic issues. Ocular myxomas (particularly lid myxomas) can present with a systemic association, however, corneal myxomas are generally an isolated lesion without cardiac or other tissue-related myxomas. None of our patients had an associated finding indicative of Carney’s complex.

The eyes with corneal myxoma in our cohort had a whitish gelatinous material in the center of the cornea. This observation is consistent with the description in the literature^3–5^.

In the current study, 8 eyes were secondary myxoma and 2 eyes were primary myxoma. Previous literature on myxoma has reported 2 primary cases, and 19 secondary cases^3–19^. The pathogenesis of corneal myxoma remains controversial. In primary cases, the lesion was thought to develop via degenerative or reactive processes versus being a true neoplastic proliferation^4^. Alternately, it has been suggested that the disruption of Bowman’s layer may be an important factor in the development of secondary corneal myxoma, which was a consistent observation in all our cases^6^. Corneal myxomas tend to form anteriorly beneath the epithelium^10^. In our cases, the myxomas were all subepithelial and the epithelial changes were variable in different specimens but shared the presence of epithelial edema in 9 out of 10 cases. Hence, this was not analyzed among the different subgroups (primary and secondary). Only one case, case 3 (92 years old female presented with high IOP and whitish corneal opacification) did not show the features documented above of epithelial edema, possibly due to the markedly hyperplastic epithelium with metaplasia and keratinization. It was proposed that the destruction of Bowman’s layer plays a major factor in the formation of corneal myxoma^4^. The outcomes of our study support this observation as all cases had a completely absent Bowman's layer or Bowman's layer was focally absent above the myxoma. Generally, corneal myxomas show spindle-shaped and/or stellate cells within a loose stroma while the myxoid stroma stains strongly with Alcian blue, indicating high glycosaminoglycan content^20^. In our cases the Alcian blue staining was observed to be less intense with an average grade of 1 in the secondary-truly acquired-cases such as in association with keratoconus, while it was consistently more intense with a grade of 3 in association with the clinical diagnosis of Peter’ anomaly and in the 2 primary cases. The cellularity was consistent with the myxoid component in the corresponding cases. Belliveau et al.’s series had only 2 patients out of 6 with moderate to severe interruption of the endothelium. Endothelial disruption might jeopardize the visual prognosis by causing stromal edema and scarring^4^.

Our cohort consisted of five pediatric patients. In the previous reports in the literature, corneal myxoma was mostly present in adult patients, except in one case report^7^. Diagnosis at older ages could be explained as either being late presentation or secondary myxoma developing subsequent to other ocular pathology.

It is clinically difficult to differentiate between primary and secondary corneal myxoma as a whitish corneal lesion is the usual presenting feature of both types and the histopathological features are similar. However, in this study we elected to separate the-truly acquired-secondary acquired myxomas, which were less cellular and stained less intensely with Alcian blue from the secondary-developmental myxomas found in association with ASD. The latter group exhibited unique IHC staining differences that could be attributed to the original underlying pathology and might indicate different pathogenesis. All myxomas showed strong reactivity to vimentin confirming our diagnosis (Figs. 2, 3). On the other hand, the 2 primary myxomas and all secondary (acquired) myxomas except the one with associated keratitis did not show reactivity with SMA. In contrast, three cases of ASD had some reactivity with SMA, which is unusual for true myxomas. This finding is notable and seems to be irrelevant to the classification of corneal myxomas into secondary or primary but is likely related to pathology and etiology specific to anterior segment dysgenesis. This observation warrants further investigation including exploration of the pluripotent nature of some of the stromal cells during the early stages of corneal development, thus changing the cellular components of the developing myxomas in these cases with a higher proportion of modified myofibroblastic cells^1^.

In our study, the primary to secondary myxoma ratio was 2:8. Primary myxoma is rare ^7^. All six cases reported by Belliveau et al. were secondary corneal myxomas^4^. Most of them developed after disruption of the Bowman’s layer as a result of ocular surgery or trauma. In contrast, Hensan et al. reported a female aged 36 years old with primary myxoma^8^. Thus, our study is unique with variability in the histopathological characteristics and IHC staining properties between the 2 categories of secondary myxomas. Notably, 4 clinically presumed primary myxomas were reclassified as secondary-developmental-myxomas based on the additional histopathological diagnosis of ASD. It would be interesting to use the full data for meta-analysis to understand the pathophysiology of primary versus secondary myxoma. From the limited data we have and the observations of this study, primary myxomas were typical of mesenchymal origin with strong staining with vimentin and absent expression of the tumor cells to SMA. However, secondary myxomas related to ASD and infection did show expression of SMA. In addition, Vimentin is an intermediate filament protein expressed in mesenchymal lesions. In general, vimentin expression in the cornea is correlated to fibrosis and wound healing. In the cornea, it has been also observed related to PRK as an earlier indication for the proliferation of myofibroblasts that will eventually express SMA^21^. Conjunctival myxomas share these histopathological and immunohistochemical characteristics^22,23^.

Additionally, primary myxomas and the ones associated with ASD had typical mucinous stroma indicated by the intense staining with Alcian blue, while the secondary acquired myxomas had less intense staining^23^. This observation might yield insights for differentiating between the 2 based on the difference in the pathogenesis and mechanism of development of these lesions. Furthermore, it may support the theory that secondary truly acquired myxomas might represent myxomatous degeneration rather than a true stromal proliferation in the cornea. This might explain the lower association between ocular myxomas and Carney’s complex which carries the risk of similarly associated cardiac myxomas. These lesions have been studied more extensively in the heart with a detailed histopathological description of cardiac lesions representing true myxomas and those with degenerative etiology^24–26^.

Subepithelial myxomas can be treated by PTK or lamellar keratoplasty (LKP) while those with deep stromal penetration usually require PKP. Early presentation to the hospital usually shows pathology with limited stromal involvement where PTK alone might be curative. One patient in our case series underwent evisceration due to a blind, painful eye. In contrast, Belliveau et al.^4^ had three out of six eyes with myxoma.

In an institution with an estimated 11,614 PKP and LKP performed by experienced surgeons during the study period, the outcomes of surgical management are more likely to be due to factors related to disease instead of service providers. Corneal surgery could help in preserving the eyes in most cases, which is an encouraging insight. In our study, recurrence was reported in one case within two months of local resection. In the broader literature, 2 out of 21 reported corneal myxoma cases experienced recurrence^5,9^. Perhaps complete removal of the lesion with PKP instead of local excision could address the issue of recurrence.

In our study, the visual outcomes were not promising in pediatric patients although they had clear grafts. This outcome could be explained by the development of amblyopia after a long period of deprivation due to corneal pathology.

This is perhaps the largest series of histopathologically-confirmed myxomatous lesions of the cornea reported for an Arab population. Phenotype information from our study would enrich the literature on this rare condition. However, the retrospective nature of this study is a limitation. Additional documentation of the clinical profile with corneal topography and anterior segment optical coherence tomography (AS-OCT) could further improve the study. Corneal myxoma is a rare disease and therefore, a prospective investigation might be impractical.

Genotype studies may be interesting to evaluate our observation of an association of corneal myxoma with anterior segment dysgenesis in children irrespective of primary or secondary lesions.

In conclusion, corneal myxoma seems to be a rare lesion that needs to be detected in the early stages. Timely surgical intervention by PKP is recommended to preserve the globe but the visual prognosis is still not very promising. The proper visual rehabilitation and management of amblyopia as early as possible is a prime factor in successful visual outcomes. Further immunohistochemical studies are needed to understand the pathogenesis of such lesions in this ocular location with particular correlation to the lesions developing in association with congenital corneal anomalies.

## Data Availability

All data generated or analyzed during this study are included in this published article.
